# Serum neutrophil gelatinase-associated lipocalin at inception of renal replacement therapy predicts survival in critically ill patients with acute kidney injury

**DOI:** 10.1186/cc8861

**Published:** 2010-02-01

**Authors:** Philipp Kümpers, Carsten Hafer, Alexander Lukasz, Ralf Lichtinghagen, Korbinian Brand, Danilo Fliser, Robert Faulhaber-Walter, Jan T Kielstein

**Affiliations:** 1 Department of Nephrology & Hypertension, Hannover Medical School, Carl-Neuberg Strasse 1, D-30625, Hannover, Germany; 2 Department of Medicine D, Division of General Internal Medicine, Nephrology, and Rheumatology, University Hospital Münster, Albert-Schweitzer-Strasse 33, 48149 Münster, Germany; 3 Department of Clinical Chemistry, Hannover Medical School, Carl-Neuberg Strasse 1, D-30625, Hannover, Germany; 4 Renal and Hypertensive Diseases, Saarland University Medical Centre, Kirrberger Straße, D-66421, Homburg/Saar, Germany

## Abstract

**Introduction:**

Neutrophil gelatinase-associated lipocalin (NGAL) is a promising novel biomarker that correlates with the severity and outcome of acute kidney injury (AKI). However, its prognostic utility during the late course of AKI, especially in patients that require renal replacement therapy (RRT) remains unknown. The aim of this study was to evaluate the predictive value of serum NGAL in patients with established AKI at inception of RRT in the intensive care unit (ICU).

**Methods:**

Serum NGAL (ELISA methodology) was measured in 109 critically ill patients with AKI at inception of RRT in 7 ICUs of a tertiary care university hospital. The primary outcome studied was 28-day mortality. Secondary outcome measures were ICU length of stay, ventilator-free days, and renal recovery at day 28.

**Results:**

There was a significant difference in serum NGAL between healthy subjects (median [interquartile range] 39.0 [37.5-42.75] ng/mL), critically ill patients with systemic inflammatory response syndrome (SIRS) (297 [184-490] ng/mL), and critically ill patients with sepsis (708 [365-1301] ng/mL; *P *< 0.0001), respectively. Multiple linear regression showed that NGAL levels were independently related to the severity of AKI and the extent of systemic inflammation. NGAL levels were higher in non-survivors (430 [303-942] ng/mL) compared to survivors (298 [159-506] ng/mL; *P *= 0.004). Consistently, Cox proportional hazards regression analysis identified NGAL as a strong independent predictor for 28-day survival (hazard ratio 1.6 (95% confidence interval [CI] 1.15 - 2.23), *P *= 0.005).

**Conclusions:**

This is the first prospective evaluation of serum NGAL as an outcome-specific biomarker in critically ill patients at initiation of RRT. The results from this study indicate that serum NGAL is as an independent predictor of 28-day mortality in ICU patients with dialysis-dependent AKI.

## Introduction

Acute kidney injury (AKI) is a frequent complication in critically ill patients and is associated with an excess mortality [1-4]. AKI requiring renal replacement therapy (RRT) affects approximately 6% of critically ill patients and results in a hospital mortality of 45 to 60% [4-6]. Outcome prediction in this selected high-risk collective is challenging due to the lack of appropriate biomarkers and the limited value of severity-of-illness scoring systems [7-10]. Thus, the identification of outcome-specific biomarkers in this patient population is a major goal in critical care nephrology.

In experimental and clinical studies, neutrophil gelatinase-associated lipocalin (NGAL) is one of the most frequently investigated and most promising biomarkers for the early diagnosis of AKI. In fact, NGAL (also known as lipocalin 2 or *lcn2*) was found to be an excellent biomarker for the early detection of AKI in the emergency department [11], after exposure to radio-contrast media [12-14], and following cardiac surgery [15-19]. There is increasing evidence that NGAL is not only a marker of AKI *per se *but also a predictor of AKI severity and AKI-related outcomes such as requirement of RRT, length of hospital stay (LOS), and mortality [15,20].

However, despite its well-defined role in the early detection of AKI, little is known about the diagnostic and prognostic utility of NGAL during the clinical course in patients with established AKI. Therefore, we aimed to investigate the outcome-specific value of NGAL, measured at initiation of RRT in critically patients with severe AKI.

## Materials and methods

### Patients and study design

The present investigation is a sub-study from the Hannover Dialysis Outcome Trial (HANDOUT), a single-center randomized controlled trial comparing standard and intensified extended dialysis therapy in patients with AKI at seven intensive care units (ICUs) of our tertiary care center at the Hannover Medical School between 2003 and 2006. The protocol and main results of the HANDOUT trial (Clinical Trial ID: NCT00529139) have been published recently [21].

Serum samples for quantification of NGAL were available from 109 patients (Table 1). All patients were treated with extended dialysis, using the GENIUS™ dialysis system (Fresenius Medical Care, Bad Homburg, Germany) [22] with high-flux polysulphone dialyzers (F60S, 1.3 m^2^, Fresenius Medical Care, Bad Homburg, Germany).

**Table 1:** Baseline characteristics at initiation of RRT

Variable	Total	non-SIRS/sepsis	SIRS	Sepsis	*P *value
**Number of patients **(n; %)	109 (100)	25 (22.9)	53 (48.6)	31 (28.4)	
**Age **(years, median (IQR))	51 (40-61)	56 (47-68)	51 (39-60)	48 (37-61)	0.086
**Female sex **(n;%)	42 (38.5)	5 (20.0)	25 (47.2)	12 (38.7)	0.071
**LOS before start of RRT **(median (IQR))	2 (1-5)	3 (2-5)	2 (1-7)	2 (1-5)	0.426
**APACHE II score **(median (IQR))	34 (26-36)	34 (27-36)	30 (24-35)	35 (29-40)	0.028
**SOFA score **(median (IQR))	16 (13-18)	15 (13-17)	14 (12-17)	17 (16-19)	0.011
**Cardiovascular variable**	4 (1-4)	3 (1-4)	3 (1-4)	4 (3-4)	0.008
**Respiratory variable**	2 (2-3)	2 (2-3)	2 (1-3)	3 (2-4)	0.005
**Coagulation variable**	1 (1-3)	1 (1-3)	1 (1-3)	3 (1-3)	0.567
**Liver variable**	1 (1-2)	1 (1-2)	2 (1-2)	1 (1-2)	0.858
**Renal variable**	3 (2-4)	3 (2-4)	3 (2-4)	3 (2-4)	0.517
**CNS variable**	4 (3-4)	4 (4-4)	4 (2-4)	4 (4-4)	0.009
**Indication for RRT**					
**CCR/eGFR loss >30% **(n;%)	100 (91.7)	23 (92.0)	49 (92.5)	28 (90.3)	0.942
**Oliguria **(n;%)	78 (71.6)	18 (72.0)	36 (37.9)	24 (77.4)	0.647
**Severe acidosis **(n;%)	10 (9.2)	0 (0)	5 (9.4)	5 (16.5)	0.115
**Hyperkalemia **(n;%)	6 (5.5)	0 (0)	3 (5.7)	3 (9.7)	0.287
**RIFLE class**					0.947
**Risk **(n;%)	9 (8.3)	2 (8.0)	5 (9.4)	2 (6.5)	
**Injury **(n;%)	13 (11.9)	2 (8.0)	7 (13.2)	4 (12.9)	
**Failure **(n;%)	87 (79.8)	21 (84.0)	41 (77.4)	25 (80.6)	
**Laboratory data **(median (IQR))					
**CRP **(mg/L)	138 (59-204)	113 (60-155)	67 (36-176)	202 (138-303	<0.001
**Creatinine **(μmol/L)	236 (185-313)	277 (189-350)	250 (191-348)	203 (152-257)	0.475
**Cystatin C **(mg/L)	1.82 (1.37-2.68)	1.81 (1.47-2.4)	1.82 (1.41-2.93)	1.84 (1.27-2.47)	0.676
**NGAL **(ng/mL)	364 (196-582)	297 (184-490)	315 (161-455)	708 (365-1301)	0.001

APACHE II score = Acute Physiology And Chronic Health Evaluation score; CCR = creatinine clearance; CNS = central nervous system; CRP = C reactive protein; eGFR = estimated glomerular filtration rate; IQR = interquartile range; LOS = length of stay in the intensive care unit; NGAL = neutrophil gelatinase-associated lipocalin; RIFLE = a newly developed international consensus classification for acute kidney injury, that defines three grades of severity - risk (class R), injury (class I) and failure (class F); RRT = renal replacement therapy; SOFA = Sequential Organ Failure Assessment score; SIRS = systemic inflammatory response syndrome.

Inclusion criteria were AKI with RRT dependence indicated by a loss of kidney function of more than 30% calculated estimated glomerular fraction rate (eGFR) with either the Modification of Diet in Renal Disease (MDRD), Cockroft-Gault equation or cystatin C GFR within 48 hours prior to inclusion and oliguria/anuria (less than 30 mL/h for more than six hours prior to inclusion or hyperkalaemia more than 6.5 mmol/L) or severe acidosis with pH below 7.15. Urine output was determined under optimized conditions (corrected volume status, adequate titration of vasopressors, and after an unavailing trial of loop diuretics). Exclusion criteria were pre-existing chronic kidney disease as defined as eGFR less than 50 mL/min or plasma creatinine concentration above 1.7 mg/dL (above 150 μmol/L) more than 10 days prior to initiation of the first RRT.

Enrollment was performed in a randomized consecutive fashion after obtaining written informed consent from the patients or their legal representatives. If the patient was recovering and able to communicate, he or she was informed of the study purpose and consent was required to further maintain status as a study participant. The study was performed in accordance with the declaration of Helsinki and approved by the institutional review board.

Routine chemistry tests and physiological parameters, including Sequential Organ Failure Assessment (SOFA) score [23] and Acute Physiology and Chronic Health Evaluation II (APACHE II) score [24] were obtained for each patient immediately before initiation of RRT. The presence of sepsis was defined according to the SCCM/ESICM/ACCP/ATS/SIS International Sepsis Definitions (Two or more of the following findings: body temperature < 36°C or > 38°C, heart rate > 100 beats per minute, respiratory rate > 20 breaths per minute, or white blood cell count < 4,000 cells/mm^3 ^or > 12,000 cells/mm^3 ^in addition to suspected or proven infection) [25]. AKI was classified *post-hoc *by means of the RIFLE (risk of renal failure, injury to kidney, failure of kidney function, loss of kidney function and end-stage renal failure) criteria at initiation of RRT [26].

### Sampling and quantification of NGAL

Serum cystatin C and serum C reactive protein (CRP) levels were determined by routine methods in the department of clinical chemistry at Hannover Medical School. Serum samples for quantification of NGAL were obtained for each patient immediately before initiation of RRT, immediately centrifuged at 3000 g for 10 minutes, divided into aliquots and stored at -80°C. NGAL was quantified in a blinded fashion by ELISA (NGAL Rapid ELISA Kit CE IVD [Cat.No. KIT 037], BioPorto, Gentofte, Denmark) according to the manufacturer's instructions [27]. The serum concentration (median (interquartile range (IQR))) of NGAL in 10 apparently healthy volunteers was 39 (37.5 to 42.8) ng/mL.

### Outcome definitions

Survival after 28 days was calculated from the day of first dialysis to death from any cause. Patients who survived to day 28 were censored at day 28. Ventilator-free days (VFDs) were defined as the number of days between successful weaning from mechanical ventilation and day 28 after study enrollment. VFDs were 0 if the patient died before day 28 or required mechanical ventilation for 28 days or more. ICU-free days were defined as the number of days between successful transfer to a normal ward and day 28 after study enrollment. ICU-free days were 0 if the patient died before day 28 or stayed in the ICU for 28 days or more. Renal recovery was defined as no need for RRT at day 28 after study enrollment.

### Statistical analysis

Continuous variables are expressed as medians with corresponding 25^th ^and 75^th ^percentiles (IQR) and were compared using the Mann-Whitney rank sum test or the Kruskal Wallis one-way analysis of variance (ANOVA). Categorical variables were compared using the chi-squared test. Correlations between variables were assessed by the Spearman rank correlation coefficient. To identify predictors of mortality, Cox's proportional hazards regression analysis was performed using backward elimination (Wald's test). Simple and multiple linear regression analysis was performed to identify which variables best predict NGAL. Similarly, linear regression and binary logistic regression models were used to identify predictors of VFDs, ICU-free days, and renal recovery, respectively. To ful?ll the assumptions needed for the analysis, logarithmic transformation of SOFA score, single SOFA score items, APACHE II score, CRP, and NGAL was performed. The distribution of the time-to-event variables were estimated using the Kaplan-Meier method with log-rank testing. Receiver operator characteristic (ROC) curves were used to detect optimal cut-off values for NGAL. Contingency table-derived data and likelihood ratios were calculated using the StatPages website [28]. All tests were two-sided and significance was accepted at *P *< 0.05. Data analysis was performed using SPSS (SPSS Inc, Chicago, IL, USA). Figures were prepared using the GraphPad Prism (GraphPad Prism Software Inc, San Diego, CA, USA).

## Results

### Patient characteristics

Patients were grouped as non-systemic inflammatory response syndrome (SIRS)/sepsis, SIRS, and sepsis according to the International Sepsis Definitions [25]. Patient groups were comparable with respect to baseline demographics, LOS in the ICU before start of RRT, indications for RRT, and the proportion of RIFLE categories (Table 1). Patients with sepsis had moderately higher SOFA scores that resulted from more severe cardiovascular and respiratory impairment compared with patients with either non-SIRS/sepsis, or SIRS, respectively. Consistently, septic patients had poorer 28-day survival (Table 2). However, secondary outcomes, such as VFDs, ICU-free days, or renal recovery, were not different between the groups (Table 2).

**Table 2:** Outcomes at day 28 after initiation of RRT

Variable	Total	non-SIRS/sepsis	SIRS	Sepsis	*P *value
**Mortality **(n;%)	41 (37.6)	6 (24.9)	15 (28.3)	20 (64.5)	0.001
**VFDs **(mean ± SD)	7.4 ± 10.3	5.5 ± 9.5	8.3 ± 10.7	7.2 ± 10.1	0.580
**ICU-free days**^† ^(mean ± SD)	5.2 ± 8.4	4.6 ± 8.3	5.4 ± 8.4	5.4 ± 8.8	0.738
**Renal recovery**^‡ ^(n;%)	45 (66.2)	13 (68.4)	26 (68.4)	6 (54.5)	0.673

ICU = intensive care unit; SD = standard deviation; SIRS = systemic inflammatory response syndrome; VFDs = ventilator-free days (defined as the number of days between successful weaning from mechanical ventilation and day 28 after study enrollment).

† ICU-free days were defined as the number of days between successful transfer to a normal ward and day 28 after study enrollment; ‡ Renal recovery was defined as no need for RRT at day 28 after study enrollment.

### Predictors of serum NGAL levels at baseline

**Figure 1:**
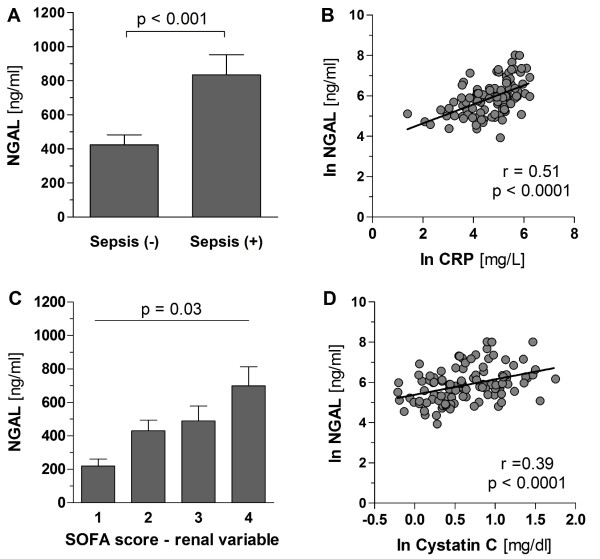
**Correlation of NGAL serum levels**. **(a and c)**. Correlation of NGAL serum levels with sepsis and acute kidney injury (AKI). Bar charts (mean ± standard error of the mean) showing serum neutrophil gelatinase-associated lipocalin (NGAL) levels of critically ill patients with AKI at inception of renal replacement therapy (RRT) stratified by **(a) **the presence (n = 31) or absence (n = 78) of sepsis as according to the SCCM/ESICM/ACCP/ATS/SIS International Sepsis Definitions [25], or **(c) **stratified by the renal variable from the Sequential Organ Failure Assessment (SOFA) score (1 point (n = 9), 2 points (n = 27), 3 points (n = 28), 4 points (n = 45)). **(b and d) **Scatter plot showing the correlation of serum NGAL concentrations with **(c) **C reactive protein (CRP) levels, and **(d) **serum cystatin C levels in critically ill patients at initiation of RRT (n = 109).

Critically ill patients had significantly higher serum NGAL levels at initiation of RRT compared with healthy controls (364 (196 to 582) ng/mL vs. 39.0 (37.5 to 42.75) ng/mL; *P *< 0.0001). To identify which mediator or index is best related to NGAL, we initially carried out a linear regression analysis for all baseline variables included in Table 1. All variables found to be statistically significant at a 10% level in the simple model were then included in a multiple linear regression model using backward elimination. Using NGAL as the dependent variable, the presence of sepsis, the renal variables from the SOFA score, CRP, and serum cystatin C were independently related to NGAL levels at initiation of RRT (Table 3). As expected, serum NGAL concentrations were significantly higher in patients with sepsis compared with patients without sepsis (834 ± 118 ng/mL (mean ± standard error of the mean (SEM)) vs. 425 ± 57 ng/mL; *P *< 0.001; Figure 1). No difference was found between patients with SIRS and non-SIRS/sepsis patients (Table 1). Furthermore, serum NGAL steadily increased across groups when stratified by the renal variable from the SOFA score (1 point: 220 ± 42, 2 points: 430 ± 63, 3 points: 489 ± 89, 4 points: 700 ± 114; *P *= 0.03 by non-parametric ANOVA; Figure 1). The relation of NGAL with these variables was further illustrated by linear correlation analysis, showing that NGAL levels correlated with CRP (r = 0.51; *P *< 0.0001), and cystatin C (r = 0.39; *P *< 0.0001) levels, respectively (Figure 1).

**Table 3:** Simple and multiple linear regression analysis with neutrophil gelatinase-associated lipocalin as dependent variable

	Simple model		Multiple model	
**Variable**	***B***	***P *value**	***β***	***P *value**

**Sepsis **(yes/no)	0.37	<0.0001	0.33	< 0.001
RIFLE category failure*	0.23	0.018	0.04	0.693
**ln SOFA - total**	0.16	0.098	0.03	0.738
**ln SOFA - **respiratory variable	0.17	0.075	0.13	0.140
**ln SOFA - **renal variable	0.22	0.020	0.23	0.007
**ln CRP**	0.40	<0.0001	0.21	0.031
**ln cystatin C**	0.34	<0.0001	0.26	0.005

Multiple linear regression analysis using neutrophil gelatinase-associated lipocalin as the dependent variable. Variables found to be statistical significant at a *P *value less than 0.1 in the simple model were included into the multiple linear regression analysis using stepwise backward elimination. Variables that were not normally distributed were log transformed. (*β *denotes standardized regression coefficient); A two-sided *P *value of less than 0.05 was considered statistically significant in the multiple model. * *β *for categorical variables is only displayed in case of multivariate significance.

CRP = C reactive protein; RIFLE = a newly developed international consensus classification for acute kidney injury, that defines three grades of severity - risk (class R), injury (class I) and failure (class F); SOFA = Sequential Organ Failure Assessment score.

### NGAL levels during follow-up

Serum samples obtained at day 14 were available from 61 patients. After initiation of RRT, NGAL levels declined significantly until day 14 (364 (196 to 582) ng/mL vs. 206 (93 to 349) ng/mL; *P *< 0.0001). However, median NGAL levels were not different between survivors (176 (89 to 338) ng/mL, n = 49) and non-survivors (281 (138 to 726) ng/mL, n = 12; *P *= 0.114), respectively.

### Predictors of 28-day mortality at inception of RRT

NGAL levels were higher in non-survivors (430 (303 to 942) ng/mL) compared with survivors (298 (159 to 506) ng/mL; *P *= 0.004). To test whether pre-RRT levels of NGAL predict 28-day mortality, we initially performed univariate Cox proportional hazards analyses, incorporating multiple demographic, clinical and laboratory variables at start of RRT (Table 4). All variables found to be statistically significant at a 10% level in the univariate analysis (sepsis, APACHE II score, SOFA score, CRP, and NGAL) were subjected to multivariate Cox regression analysis. As a result, the SOFA score (*P *= 0.004), and serum NGAL (*P *= 0.005) were identified as independent predictors of 28-day mortality. Essentially, the same results were obtained in an adjusted model incorporating treatment intensity of RRT (standard vs. intensified extended dialysis in the original HANDOUT trial).

**Table 4:** Predictors of 28-day mortality using Cox proportional hazards regression analysis

	Univariate		Multivariate	
	
Variables	HR (95% CI)	*P *value	HR (95% CI)	*P *value
**Age **(per 10 years)	0.97 (0.79-1.20)	0.791		
**Sex **(female)	0.76 (0.40-1.45)	0.401		
**SIRS**^†^	1.22 (0.48-3.16)	0.675		
**Sepsis**^†^	3.54 (1.42-8.82)	0.007		
RIFLE category*	--	0.381		
**ln SOFA - **renal variable (per 1 SD ↑)	1.05 (0.66-1.67)	0.846		
**LOS before start of RRT **(per day)	1.00 (0.96-1.04)	0.889		
**ln APACHE II score **(per 1 SD ↑)	1.72 (1.15-2.56)	0.008		
**ln total SOFA score **(per 1 SD ↑)	1.79 (1.28-2.50)	<0.001	1.62 (1.17-2.25)	0.004
**ln CRP **(per 1 SD ↑)	1.71 (1.13-2.58)	0.012		
**ln cystatin C **(per 1 SD ↑)	1.07 (0.77-1.50)	0.679		
**ln NGAL **(per 1 SD ↑)	1.79 (1.28-2.51)	<0.001	1.60 (1.15-2.23)	0.005

Estimated hazard ratios (HR), 95% confidence intervals (CI), and *P *values were calculated by Cox regression analyses (backward elimination). Variables found to be statistical significant at a *P *value of less than 0.1 in the univariate model were included into the multivariate model using stepwise backward elimination. A two-sided *P *value of less than 0.05 was considered statistically significant in the multivariate model. Variables that were not normally distributed were log transformed; hazard ratios refer to 1 standard deviation (SD) in the log scale in these variables. † For SIRS and sepsis, the non-SIRS/sepsis group was set as the reference group. *HR (95% CI) for categorical variables is only displayed in case of multivariate significance.

APACHE II score = Acute Physiology And Chronic Health Evaluation score; CRP = C reactive protein; LOS = length of stay in the intensive care unit; NGAL = neutrophil gelatinase-associated lipocalin; RIFLE = a newly developed international consensus classification for acute kidney injury, that defines three grades of severity - risk (class R), injury (class I) and failure (class F); RRT = renal replacement therapy; SOFA = Sequential Organ Failure Assessment score; SIRS = systemic inflammatory response syndrome.

**Figure 2:**
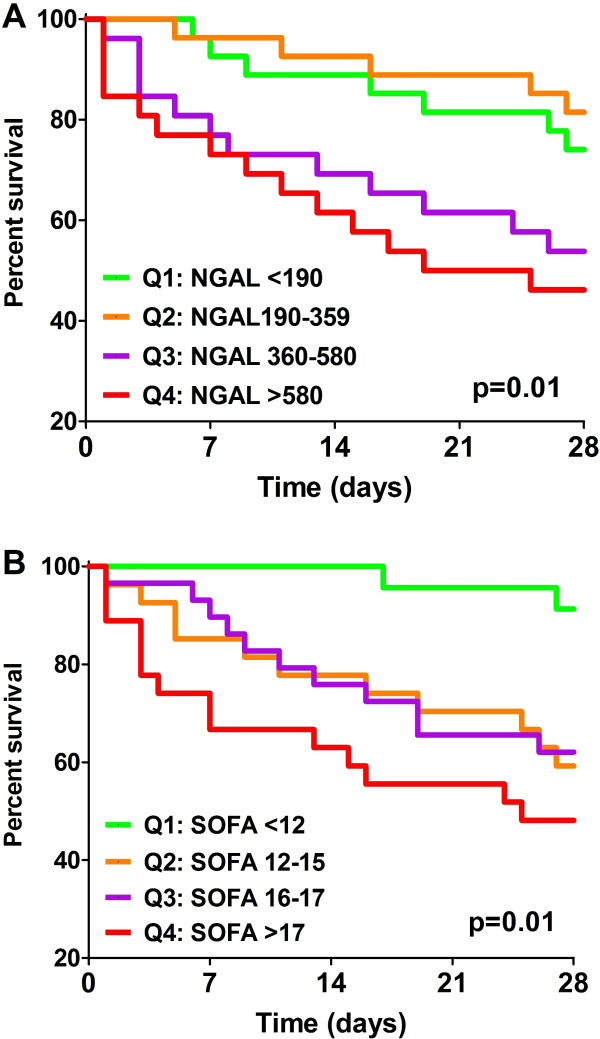
**Survival to day 28 according to serum NGAL**. Kaplan-Meier curves of 28-day survival stratified to **(a) **neutrophil gelatinase-associated lipocalin (NGAL) quartiles (Q) (Log-rank (Mantel-Cox) *P *= 0.01; Log-rank test for trend *P *= 0.003), and **(b) **Sequential Organ Failure Assessment (SOFA) quartiles (total SOFA), respectively (Log-rank (Mantel-Cox) *P *= 0.01; Log-rank test for trend *P *= 0.002) at inception of renal replacement therapy in critically ill patients with acute kidney injury (n = 109).

When visualized by Kaplan-Meier curves, mortality was no different between NGAL quartile (Q) 1 and Q2, but steadily increased among Q3 and Q4 (Log-rank (Mantel-Cox) *P *= 0.01; Log-rank test for trend *P *= 0.003; Figure 2a). Similarly, 28-day mortality was low in the SOFA Q1, intermediate in Q2 and Q3, and high in SOFA Q4, respectively (Log-rank (Mantel-Cox) *P *= 0.01; Log-rank test for trend *P *= 0.002; Figure 2b).

### Serum NGAL test characteristics at various cut-off values at initiation of RRT

**Figure 3:**
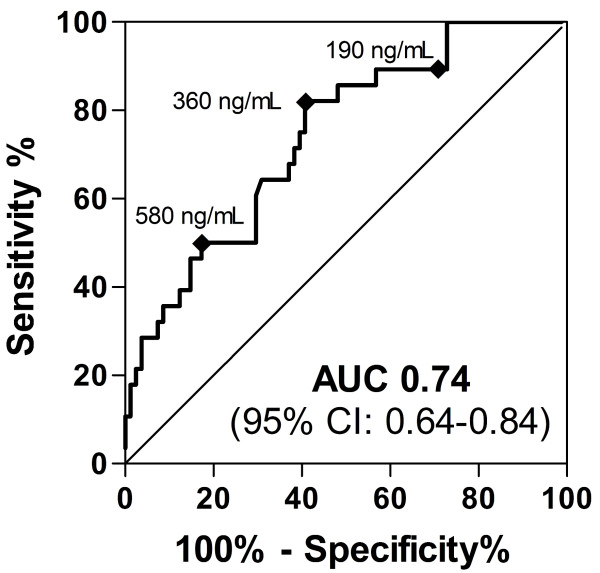
**Serum NGAL test characteristics at various cut-off values at initiation of RRT**. Receiver-operator characteristic (ROC) curve showing the prognostic sensitivity and specificity of serum neutrophil gelatinase-associated lipocalin (NGAL) at initiation of renal replacement therapy (RRT) with regard to 14-day mortality (area under the curve (AUC) 0.74 (95% confidence interval (CI) 0.64 to 0.84) *P *< 0.0002). Cuboids indicate the cut-off values between NGAL quartiles (see also Figure 2 and Table 5).

To assess the utility of NGAL measurements at varying cut-off values to predict mortality, a conventional ROC curve was generated and the area under the curve (AUC) calculated (Figure 3). Table 5 lists the derived sensitivities, specificities, and predictive values at different cut-off concentrations (according to NGAL Qs). For serum NGAL at initiation of RRT, sensitivity and specificity were optimal at the 360 ng/mL cut-off, with an AUC of 0.74 (95% confidence interval (CI) 0.64 to 0.84) for the prediction of death before day 14 (Figure 2).

**Table 5:** Serum NGAL test characteristics at various cut-off values at initiation of renal replacement therapy

NGAL cut-off	OR	**Sensitivity **(%)	**Specificity **(%)	**NPV **(%)	**PPV **(%)
**≥ 580 ng/mL**	4.79	50.0	82.7	82.7	50.0
**≥ 360 ng/mL**	6.69	82.1	59.2	90.6	41.1
**≥ 190 ng/mL**	3.31	89.3	28,4	88.5	30.1

NGAL = neutrophil gelatinase-associated lipocalin; NPV = negative predictive value; OR = odds ratio; PPV = positive predictive value.

### Secondary outcome measures

Renal recovery (defined as no need for dialysis in survivors at the end of the study period) was not associated with serum NGAL levels as determined by binary logistic regression analysis (*P *= 0.84). The same was true for VFDs (*P *= 0.75) and for ICU-free days (*P *= 0.574), as shown by linear regression analysis, respectively.

## Discussion

This is the first study investigating the predictive value of NGAL for the outcome of critically ill patients with established AKI at initiation of RRT. Elevated serum NGAL is independently related to the severity of AKI (cystatin C and renal SOFA), but also to the presence of sepsis and the extent of systemic inflammation (CRP). Serum NGAL was higher in critically ill patients who died compared with patients who survived during the study period. Consistently, Cox proportional hazards regression analysis identified NGAL as an independent predictor for 28-day survival in our cohort.

Gene expression studies in AKI have demonstrated that NGAL is highly up-regulated in the thick ascending limb of Henle's loop and the collecting ducts [29]. The resultant synthesis of NGAL protein in the distal nephron and secretion into the urine appears to comprise the major fraction of urinary NGAL [29,30]. It is assumed that urinary NGAL is probably more reflective of local renal injury [29,31], because systemic NGAL in serum or plasma may, at least in part, be derived from stressed immune cells and injured epithelial cells of the lungs and the gastrointestinal tract [32,33]. Wheeler and colleagues demonstrated that serum NGAL concentrations were increased at 24 and 72 hours after ICU admission in children who developed AKI compared with children who did not develop AKI [20]. Of note, in the same cohort, serum NGAL was significantly increased in critically ill children with sepsis compared with critically ill children without sepsis.

In the present study, both, the presence of sepsis as well as the severity of AKI were independently associated with increased serum NGAL concentrations in a multiple linear regression analysis model. Thus we assume that plasma NGAL not only depicts AKI, but also reflects the severity of systemic inflammation. In line with this interpretation, increased NGAL concentrations have been found in the blood of patients with acute bacterial infections and during experimental human endotoxemia [34,35]. Unfortunately, urinary NGAL could not be determined because most of the patients presented with oliguria/anuria at baseline. Thus, we cannot provide any comparison between the local and systemic NGAL pool in the current study. However, to date, there is no conclusive evidence for urine NGAL being superior to plasma NGAL [17,18,20,36,37].

Recent studies have demonstrated the utility of early NGAL measurements for predicting clinical outcomes of AKI. In adults and children undergoing cardiac surgery, plasma NGAL levels strongly correlated with duration and severity of AKI, time on mechanical ventilation, LOS, and mortality [17,36,37]. However, the predictive utility of NGAL throughout the course of critical illness in patients with already established AKI is poorly characterized and has not been tested specifically at the initiation of RRT. In the current study, serum NGAL was identified as an independent predictor of 28-day mortality in the multivariate Cox model. In addition to serum NGAL, only the (total) SOFA score remained significant at the multivariate level. A ROC analysis suggested that a serum NGAL cutoff value of 360 ng/mL at initiation of RRT is highly sensitive to discriminate between survivors and non-survivors. Given a rather poor positive predictive value of 41%, a serum NGAL level of 360 ng/mL or greater probably does not serve as a robust biomarker for predicting mortality in this cohort of patients. However, NGAL levels of less than 360 ng/mL may have the potential to predict survival with a negative predictive value of 90%. If validated in a larger cohort, our observations suggest a pivotal role for serum NGAL as an outcome-specific marker in critically ill patients with multiple organ dysfunction syndrome.

This study has important limitations. First, it is a single-center cohort study of adult patients without chronic kidney disease. Our results, although of clear statistical significance, will certainly need to be validated in a larger trial, including patients with pre-existing chronic kidney disease and comorbid conditions that normally accumulate with impaired renal function. Moreover, the original study was performed between 2003 and 2006. It has been indicated that long-term storage might destabilize NGAL [38]. Thus, extrapolating our result to other ICU populations requires caution. Second, NGAL did not predict secondary outcomes, such as renal recovery, VFDs, or ICU-free days. This discrepancy may have resulted from the relatively small number of VFDs and ICU-free days. Unfortunately, the follow-up was only 28 days in the original HANDOUT trial. Moreover, weaning from mechanical ventilation was not guided by standardized protocol. The same was true for discharge from ICU. Third, the NGAL cutoff in the current study was somewhat higher than in most of the aforementioned AKI studies. However, this is not surprising because most of our patients already presented with severe AKI (RIFLE category failure). Finally, NGAL was not different between survivors and non-survivors at day 14. However, as a 25 kDa protein NGAL is most likely to be cleared by dialysis. Thus, the quantification of NGAL after the start of RRT will probably yield invalid results.

## Conclusions

Outcome prediction in dialysis-dependent ICU patients is hampered by the limited value of severity-of-illness scoring systems [7-10]. Thus, the identification of outcome-specific biomarkers in this patient population is a major goal in critical care nephrology. The results from this study indicate that serum NGAL, measured at initiation of RRT, is as an independent predictor of 28-day mortality in ICU patients with AKI. Given the lack of appropriate biomarkers in these patients, serum NGAL may serve as a novel outcome-specific marker in intensive care medicine and critical care nephrology.

## Key messages

• This is the first prospective evaluation of serum NGAL as an outcome-specific biomarker in critically ill patients at initiation of RRT.

• Serum NGAL levels at initiation of RRT were independently related to the severity of AKI and the extent of systemic inflammation.

• The results from this study indicate that serum NGAL is an independent predictor of 28-day mortality in ICU patients with dialysis-dependent AKI.

## Abbreviations

AKI: acute kidney injury; ANOVA: analysis of variance; APACHE II: Physiology and Chronic Health Evaluation II; AUC: area under the curve; CI: confidence interval; CRP: C-reactive protein; eGFR: estimated glomerular filtration rate; ELISA: enzyme linked immunosorbent assay; HANDOUT: Hannover Dialysis Outcome Trial; ICU: intensive care unit; IQR: interquartile range; LOS: length of stay; MDRD: Modification of Diet in Renal Disease; NGAL: neutrophil gelatinase-associated lipocalin; Q: quartile; RIFLE: RIFLE denotes a newly developed international consensus classification for acute kidney injury, that defines three grades of severity - risk (class R), injury (class I), failure (class F), loss (class L), and end-stage renal disease (class E); ROC: receiver operator characteristic; RRT: renal replacement therapy; SEM: standard error of the mean; SIRS: systemic inflammatory response syndrome; SOFA: Sequential Organ Failure Assessment; VFDs: ventilator-free days.

## Competing interests

The authors declare that they have no competing interests.

## Authors' contributions

PK had the initial idea for the study, supervised the measurements, analyzed the results, prepared the figures and wrote the manuscript. AL, RL, and KB performed the measurements, and contributed to the manuscript. CH, RF, and DF designed, conducted, and supervised the original HANDOUT trial, and reviewed the manuscript. JTK designed, conducted, and supervised the original HANDOUT trial, and contributed to the manuscript. PK and CH contributed equally to the work and are both considered first authors. JTK and RF contributed equally to the work and are both considered senior authors. All authors read and approved the final manuscript.
